# Quality assurance within the context of genome diagnostics (a german perspective)

**DOI:** 10.1515/medgen-2023-2028

**Published:** 2023-06-13

**Authors:** Kraft Florian, Anna Benet-Pagès, Daniel Berner, Anna Teubert, Sebastian Eck, Norbert Arnold, Peter Bauer, Matthias Begemann, Marc Sturm, Stephanie Kleinle, Tobias B. Haack, Thomas Eggermann

**Affiliations:** Medizinische Fakultät der RWTH Aachen Institut für Humangenetik und Genommedizin Aachen Deutschland; Institut für Neurogenomik Helmholtz Zentrum München Neuherberg Deutschland; MVZ genetikum GmbH Neu-Ulm Deutschland; amedes genetics Hannover Deutschland; Bionano 92121 San Diego USA; Universitätsklinikum Schleswig-Holstein Zentrum für familiären Brust- und Eierstockkrebs; Klinik für Gynäkologie und Geburtshilfe Kiel Deutschland; CENTOGENE N.V. Rostock Deutschland; Medizinische Fakultät der RWTH Aachen Institut für Humangenetik und Genommedizin Aachen Deutschland; Universität Tübingen Institut für Medizinische Genetik und Angewandte Genomik Tübingen Deutschland; Universität Tübingen Institut für Medizinische Genetik und Angewandte Genomik Tübingen Deutschland; Medizinisch Genetisches Zentrum München Deutschland; Medizinische Fakultät der RWTH Aachen Institut für Humangenetik und Genommedizin Pauwelsstr. 30 52074 Aachen Deutschland

**Keywords:** quality assurance, next generation sequencing, variant classifications, data processing

## Abstract

The rapid and dynamic implementation of Next-Generation Sequencing (NGS)-based assays has revolutionized genetic testing, and in the near future, nearly all molecular alterations of the human genome will be diagnosable via massive parallel sequencing. While this progress will further corroborate the central role of human genetics in the multidisciplinary management of patients with genetic disorders, it must be accompanied by quality assurance measures in order to allow the safe and optimal use of knowledge ascertained from genome diagnostics. To achieve this, several valuable tools and guidelines have been developed to support the quality of genome diagnostics. In this paper, authors with experience in diverse aspects of genomic analysis summarize the current status of quality assurance in genome diagnostics, with the aim of facilitating further standardization and quality improvement in one of the core competencies of the field.

## 1 Introduction

The use of Next-Generation Sequencing (NGS)-based analyses in routine human genetic diagnostics has led to a substantial increase in the detection of clinically relevant genomic changes in patients with diverse clinical presentations, and allows the comprehensive, cost-effective, and prompt clarification of genetically heterogeneous diseases. Increasingly, NGS-based findings are being used as the basis for personalized therapy approaches. The high relevance of rapid genetic diagnostics in the management of patients (and their families) is well demonstrated by the example of the care of acutely ill children (e. g. (1)). Human genetic diagnostics has thus become a central, cross-specialities discipline, in which the core human genetic competence of variant interpretation is an essential component of multidisciplinary patient care.

Besides the aforementioned increasing demand for genetic testing, the dynamics of NGS-based analytics is driven by rapid technological advances in both the wet laboratory procedures and in the bioinformatic processing and subsequent evaluation of NGS data. While at the beginning of the use of NGS in routine diagnostics, indication-specific analyses were primarily performed using multigene panels, the use of exome sequencing (ES) is now rapidly increasing, at least as a technical basis for data evaluation. Depending on the given clinical question, the generated ES data are evaluated in either a gene-specific manner, with regard to disease-causing changes for selected phenotypes, or in an exome-wide manner in the case of heterogeneous or non-specific clinical presentations. With both enrichment and evaluation strategies, single nucleotide variants and insertion-deletion variants (SNVs/InDels) as well as copy number deviations (CNVs) can be detected. However, for methodological reasons, other types of variants, such as repeat expansions (REs) or structural variants, such as translocations and inversions (subsequently referred to as SV), largely escape detection, and at present, these must be specifically investigated using other methods. Typically, coverage of the analysis target regions is not homogeneous, and this requires appropriate consideration during evaluation and reporting (type A-C tests (2)). Further potential problems in terms of evaluation arise from pseudogenes and transcript variants. To address these challenges, international projects, such as MANE (Matched Annotation from the NCBI and EMBL-EBI), which aim to integrate transcript annotation and define a genome-wide set of reference transcripts, are necessary initiatives (3).

The (further) development and diagnostic implementation of methodological approaches such as genome sequencing (GS) (overview: (4)) contribute to the improved detection of various types of pathogenetically relevant changes, as complemented in part by methods such as long-read sequencing and transcription analyses. Besides the implementation of bioinformatic algorithms for the detection of additional variant types, the added value of GS compared to ES results in particular from the expansion of the investigation beyond the target region of the protein-coding exons. At writing, insufficient data on the frequency and annotation of variants in non-coding regions renders GS challenging for diagnostic laboratories.

The complexity of NGS analyses, including their methodological limitations and technical dynamics, together with the pressing and ever increasing need for human genetic diagnostics, renders the formulation of quality standards necessary. In the context of NGS diagnostics, a laboratory must fulfil this requirement in order to offer a state-of-the-art and high-quality international standard that ensures maximum patient benefit.

Below, suggestions that are intended to contribute to standardized, quality-assured NGS diagnostics in Germany are presented, summarising existing international guidelines and recommendations on quality assurance.

The present authors refer in particular to the recommendations published by EuroGentest. These recommendations were formulated as early as 2016, and were largely adopted by the S1 guideline “Molecular genetic diagnostics with high-throughput germline methods, for example with next-generation sequencing (2018)” of the German Society of Human Genetics (5). They have recently been expanded to include the diagnostic use of GS (2, 5, 6), and are applicable to both constitutional and somatic NGS diagnostics within the field of human genetics.

The following discussion is based on the current structure of DIN EN ISO 15189 (subsequently referred to as ISO 15189) as a formal guideline for quality assurance, and all explanations are aligned—as far as possible—with the aforementioned recommendations of EuroGentest (“EuroGen recom.” (6)). Additional requirements are also taken into account, such as the guidelines of the German Medical Association (RiLiBÄK: https://www.bundesaerztekammer.de/fileadmin/user_upload/BAEK/Themen/Qualitaetssicherung/_Bek_BAEK_RiLi_QS_laboratoriumsmediziner_Untersuchungen.pdf), and other relevant contributions to the topic (e. g., (7, 8)). In addition to the aforementioned documents on quality assurance for NGS analyses, other recommendations are also available, e. g., from the Global Alliance for Genomics and Health (GA4GH), the German Society for Human Genetics (GfH), the Gene Diagnostics Commission (GEKO), and the Medical Genome Initiative.

## 2 General considerations

In general, exome and genome-wide analyses should be performed when a relevant improvement in quality, efficiency, and detection rate can be expected for the clarification of the pathogenetic cause of a clinical presentation (EuroGen recom. 1 (6)). However, in the case of a specific genetic disease with a defined genetic cause, a targeted genetic examination at the beginning of the diagnostic process may be sufficient. Whenever possible, a stepwise approach should be applied to answer the specific clinical question (EuroGen recom. 5 (6)). However, since the clinical presentation of many genetic diseases can show wide variation and/or overlap, targeted molecular testing is often problematic. NGS-based diagnostic approaches offer the opportunity to capture a broad spectrum of molecular changes in a one-step approach. Nevertheless, when ordering the test, both the appropriateness of the investigation and the processing time (e. g., in the case of relevance to therapy) must be taken into account.

Furthermore, before the implementation of an analytic process, the laboratory should determine how to manage additional or incidental findings (9), particularly in the case of exome- and genome-wide procedures (EuroGen recom. 31 (6)). If necessary, this should be communicated to the referring clinician, with reference to the specifications of the commissioning physician (10). Here, suggestions from various expert bodies should be considered (e. g., the ClinGen Actionability working group, American College of Medical Genetics (11–13)).

### 2.1 Legal and normative requirements for quality assurance

With regard to legal and normative requirements, quality assurance of genome diagnostics does not differ from that required for other human genetic diagnostic methods. However, due to the volume of data generated, the aforementioned potential for additional or incidental findings, and the possible use of equipment and software solutions outside the direct control of the facility, additional regulations within the legal and normative framework are required.

In Germany, the requirements of the Genetic Diagnostics Act (GenDG; http://www.gesetze-im-internet.de/gendg/index.html) apply. In addition, the implementation of diagnostic procedures must be based on RiLiBÄK. Though accreditation according to ISO 15189 is not mandatory for genetic diagnostics in Germany, this international norm provides an additional framework. In 2022, a revision of ISO 15189 was put in force, which must be implemented from 2025 onwards, following a period of transition. Other standards that are applied by laboratories, depending on the focus of their activities, are DIN EN ISO 17020 (applicable to the field of pathology/neuropathology) and DIN EN ISO 17025 (testing and calibration laboratories working in a non-medical context). In terms of software development, IEC 62304 (for “medical device software”) can provide assistance with respect to the requirements for, and the development of, software in genetic diagnostics. In addition to these laws and standards, which largely concern genetic analyses, since 2022, Regulation (EU) 2017/746 on in-vitro diagnostics has also been mandatory.

### 2.2 Objective of genome analyses

The purpose of diagnostic genome analyses is to determine both hereditary and somatic causes of disease. This means that the analysis of different primary samples, such as blood and tissue, may be necessary, depending on the indication. In current diagnostics, “short-read” sequencing techniques with read lengths of 100–300 bp are mainly used. Depending on the characteristics of the gene of interest, these can be complemented by “long-read” sequencing with read lengths of 10–100 kb (14).

In a number of syndromic diseases, pathogenic variants occur regularly in a mosaic state (e. g., Proteus syndrome (*AKT1*), tuberous sclerosis (*TSC1*)). In diseases such as neurofibromatosis type 1, while variants in the affected tissue may be mosaic, they may not be detectable in blood. In addition, the detection of somatic variants is used as a biomarker of treatment response in oncology patients, e. g., in PARP inhibitor therapy (15). Therefore, bioinformatic pipelines and quality parameters with an adequate limit of detection (LoD) should be used (6). A comprehensive human genetic evaluation may also include the detection of variants in the mitochondrial genome. The particular characteristics of these variants, such as tissue differences in “mosaic status” (16, 17) should be taken into account. In addition to determining sensitivity and precision, validation also should include determining the LoD for the detection of heteroplasmic mitochondrial variants. The presence of NUMTs (nuclear mitochondrial DNA sequences, “mitochondrial pseudogenes”) in the nuclear genome can lead to artifacts during the detection and interpretation of variants.

In addition to the recognition of SNVs, CNVs, and SVs, bioinformatic tools also enable the detection of “runs of homozygosity (ROH)”, which can indicate the presence of uniparental (iso-)disomy (UPD), and thus an autosomal recessive or imprinting disease. In trio analyses, UPD can also be determined without ROH detection. Secondary, somatic events in the sense of clonal hematopoiesis, which lead to a copy-neutral loss of heterozygosity and may also mask pathogenic changes, can also be captured via ROH analyses (18).

In addition to the diagnostic investigation of monogenic-, oligogenic-, and (tumor) predispositions, variant detection methods are also used for the assessment of multifactorial diseases and pharmacogenetic factors. The calculation of polygenic risk scores to determine relative risks on the basis of association studies has now been introduced into human genetic diagnostics.

When performing prenatal analyses, the exclusion of maternal contamination continues to be mandatory. During a prenatal trio diagnostic investigation, this can be achieved directly from the NGS data. In the context of non-invasive prenatal diagnostics (NIPT), the analysis of cell-free fetal DNA in the maternal plasma in order to detect aneuploidy and other genomic changes must be conducted in accordance with specific quality requirements (19).

## 3 Quality assurance issues within the context of NGS-based diagnostics

The issues described below in the context of NGS-based diagnostics should be considered with reference to the quality assurance measures as specified in ISO 15189. The following considerations are also based on the guidelines of EuroGentest (6), which recommend all laboratories performing NGS to be accredited (EuroGen recom. 2). In Germany, this is not mandatory under the terms of the GenDG, but institutions that carry out genetic analyses for medical purposes must meet specific quality management requirements (GenDG § 5(1)).

### 3.1 Personnel

All personnel involved in genome diagnostics must be suitably qualified for their respective role (pre-analytics, sample collection, wet laboratory, bioinformatics, diagnostics, validation, medical evaluation), and have the appropriate training, experience, and skills (see ISO 15189:2012: 5.1). In this context, besides the legal requirements of the GenDG, the S2k guideline of the German Society for Human Genetics (GfH) (20) must also be considered. In addition to adequate staffing for the responsible supervision of technical processes, data interpretation, and reporting, this specifies the need for the integration of appropriately trained scientists (e. g., human geneticists) and physicians. An introduction into the specific working roles and ongoing training must be carried out and documented. In addition to professional qualifications (see above), individuals responsible for data evaluation and reporting must have several years of diagnostic experience in the molecular genetic analysis of sequence changes for genetically heterogeneous diseases (including raw data and variant evaluation).

### 3.2 Premises

The premises must fulfil the requirements of modern NGS diagnostics in terms of equipment, space, layout, and house-keeping (electrical system, air conditioning, statics, access-control). Working steps that are at risk of cross-contamination must be physically separated. Accordingly, the separate storage of primary samples, nucleic acids, control materials, reagents, consumables, and analysis products must be observed. In the area of data processing, all hardware components must be kept in a location that ensures the physical security of the data.

### 3.3 Consumables, equipment and software

The laboratory must have a documented procedure for the procurement and handling of equipment, including maintenance, servicing, storage, and disposal. Batches of consumables must be recorded and managed in accordance with normal standards. For all equipment, (functional) monitoring must be documented in accordance with ISO 15189. This applies to all equipment used in the laboratory processes, from small devices (e. g., pipettes) to thermal cyclers and measuring devices for quality and quantity determination of nucleic acids to the NGS platforms and associated IT systems. On implementation, all components and modules used in the procedures must also be considered in the validation process, and their validation must be documented. When appropriate, this documentation should be performed in a modular manner. Significant changes, including updates/revisions of the chemistry, the sequencing devices, or the evaluation software, must be reverified or revalidated.

Due to the high investment and maintenance costs, NGS platforms and evaluation software are sometimes used jointly by different institutions, while external providers, including commercial ones, may be used for analysis software and data storage. In principle, this cooperation is possible. However, the relevant provisions (General Data Protection Regulation, GenDG, ISO 15189) must be taken into account. For example, shared use of a device must be documented and regulated in such a way that the genetic testing facility retains complete control over the process. This includes restrictions of access to unauthorized person in order to protect the device and the correctness and security of the patient data and samples. In addition to these security measures for data and sample protection, the use of cloud-based evaluation tools must take into account the laboratory’s need to access the data at any time, and that availability and storage must be possible over the period for which documentation is required by law.

### 3.4 Preanalytics

In addition to the requirements specified in ISO 15189 (instructions for sample collection and storage), the prerequisite pre-analytical measures for NGS analyses must include consent for sample collection, implementation of the investigation, and the processing of the genetic data, as stated in § 8 of GenDG. Here, the requirements for the informed consent procedure that are specified by GEKO, including the qualification requirements for the responsible physician, must be taken into account (10).

As formulated in the ESHG guidelines for NGS diagnostics (2) and in the S1 guidelines of the GfH (5), the laboratory is tasked with providing the referring clinician with a publicly accessible list of disease-relevant genes (“target genes” in the panel offered), which defines the scope of the investigation. Information must also be provided concerning the type of genetic variant (SNVs, CNVs, SV, repeats) for which the test is validated, and the limitations of NGS diagnostics (EuroGen recom. 5, 6 (6)).

### 3.5 Implementation of NGS procedures

**Figure 1:**
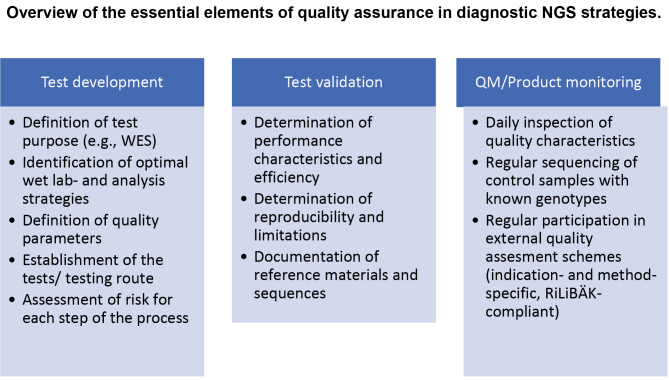
Formal levels of quality requirements in the Next Generation Sequencing process

The following sections present the various steps of the diagnostic NGS workflow, with special emphasis on quality assurance measures (Fig. 1).

#### 3.5.1 Wet laboratory

As with other molecular genetic work processes, to avoid contamination, work areas must be separated both spatially and in terms of equipment. Due to the more complex nature of these processes in NGS, including the performance of several PCR-based steps, the aforementioned separation is problematic. Deviations from strict spatial separation must therefore be monitored on site, and, if necessary, subject to a risk assessment. The estimation of risk can be facilitated by documentation of the quality parameters (see below) required in the workflow, as well as the data analysis with regard to possible contamination. In the case non-PCR-based methods, these considerations may be unnecessary.

For the wet laboratory procedures, all applied methods and their respective steps must be described in appropriate standard operating procedures. Besides the actual workflow, the documentation of analysis-relevant batches and quality-relevant data from the NGS enrichment must also be specified, and the associated parameters must be defined. This also applies to the evaluation parameters and their history (see below). If the data are documented electronically, the relevant modules must be described, with respect to documentation control, data security, and validation.

Laboratory and organizational measures for the unambiguous identification and tracking of samples must be implemented (ESHG: statement 16 (2)).

#### 3.5.2 Data processing

**Figure 2:**
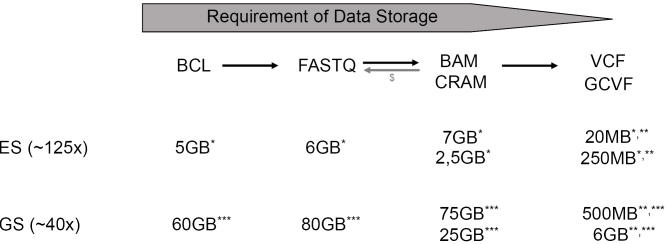
Data storage requirements in NGS diagnostics using the example of exome and genome sequencing. Data flow and exemplary ES and GS dataset sizes are shown. (* Average file size for 1 sample at 125x coverage and a read length of 2x150 bases. File size may vary slightly, depending on enrichment, compression level, and which instrument platform is used (due to different base quality score encoding). ** Average size for VCF files generated using the “GATK best practice workflow”. This can vary substantially, depending on the applied variant caller, filter strategy, and compression level. ***Average file size for 1 sample at 40x coverage and a read length of 2x150 bases. File size may vary slightly, depending on the compression level and the applied sequencer (due to different base quality score encoding). ^$^Conversion of BAM/CRAM format back to FASTQ leads a loss of information, since only the first part of the Illumina read name is overtaken in the BAM/CRAM file format. Information on reads 1 or 2 in paired-end sequencing, pass filter, and the index sequence are lost.

An NGS bioinformatic pipeline includes algorithms that process sequencing data and associated metadata, from the raw data to variant annotation and variant prioritization. This process may involve multiple software components, databases, and operating environments (hardware and operating system). In addition, diverse file types are used and generated during data analysis (Tab. 1, Fig. 2). Standard data formats, such as FASTQ, BED, SAM, BAM, CRAM, and VCF, are widely used and recommended (EuroGen recom. 17).

**Table 1:** Short-read next generation sequencing file formats. (*BAM and CRAM files also contain non-aligned sequences).

**File type**	**Content**
BCL	*binary base call*—Contains the raw sequence data
FASTQ	Sequenced reads with corresponding quality values.
BED	*Browser Extensible Data:* Generic format for specifying genomic regions as a combination of genomic coordinates and annotations.
SAM	*Sequence Alignment Map:* Information concerning reads and their mapping in the genome.
BAM*	*Binary Alignment Map:* Compressed binary version of the SAM file.
CRAM*	*Compressed Reference-oriented Alignment Map:* Highly compressed version of the SAM file.
VCF	*Variant Call Format:* Generic format for describing genetic variants.
GVCF	*Genomic VCF* format with information related to each base in the genome irrespective of the presence of a variant

The workflow for clinical NGS-based data processing comprises three main process steps: a primary analysis for generating DNA sequence data from the raw data; a secondary analysis, which includes read alignment to a reference genome and variant calling; and a tertiary analysis for variant annotation, filtering, interpretation, and reporting (7). Although the bioinformatic process for the analysis of GS does not differ substantially from that used for ES, additional algorithms are often necessary, e. g., for SV detection, repeat expansions, and annotation of non-coding regions.

##### 3.5.2.1 Primary analysis

The primary analysis includes base calling, i. e., the generation of sequence- and quality data (PHRED scores), and demultiplexing of the raw data, which are usually saved in FASTQ format. The PHRED score is an important measure for the correctness of an individual base call, and can be influenced, for example, by technical artifacts. Here, the “GATK best practice workflow”, among others, enables the partial correction of these errors by recalibrating the basic quality score (BQSR) via machine learning. Artifacts in NGS alignments are device-independent, and often result in *de novo* false positive variants. During variant filtering, systematic errors can be reduced via a comparison with data sets generated on the same sequencer. Removing adapters and trimming poor-quality bases ensures that only high-quality, optimal-length reads are used for downstream analysis.

##### 3.5.2.2 Secondary analysis

The secondary analysis is the computationally most intensive aspect of an NGS analysis. The choice of both the reference genome (GRCh37/hg19, GRCh38/hg38, T2T-CHM13) and the alignment algorithm (e. g., for DNA BWA-MEM, DRAGMAP) can have a major impact on the accuracy of complex variant calls (21). While improved variant detection and genotyping have been demonstrated for the recently published T2T-CHM13 assembly (22), its use in clinical diagnostics has not yet been adequately evaluated. A broad diagnostic application is currently precluded by the lack of transference for variant databases (e. g., ClinVar, gnomAD) to the genomic positions in the T2T-CHM13 genome. Benchmarking studies show that the use of GRCh38/hg38 improves variant detection, and generates fewer false-positive variants, compared to the use of GRCh37/hg19, and its use is therefore recommended by Eurogentest (EuroGen recom. 15 (6)). However, some databases only contain datasets for either GRCh37 or GRCh38, and in that case, the “complete” annotation of variants is only possible via a data liftover. Due to erroneously integrated duplications in the GRCh38 assembly, variant detection for several genes (e. g., *CBS*, *KCNE1*, and *CRYAA*) is worse compared to GRCh37. However, this problem can be solved by masking these areas in the reference genome (21). One approach to improving variant detection in these genes and in other genes with homologous copies in the genome (e. g., *SMN1*, *PKD1*, *NEB*, *GBA1*, *STRC*) is to use sequence graphs in the alignment algorithms (23).

Aligned sequences are stored in compressed BAM and CRAM files, together with information on their position in the genome (Tab. 1, Fig. 2). To avoid artifacts and false-positive variants, duplicates should be marked or removed, and local realignment performed. In contrast to ES, library preparations in GS are generated PCR-free when sufficient DNA is available. This reduces duplicate reads to 3–5 %, compared to 5–15 % in a typical ES procedure (24). The capture of GC-rich areas, on the other hand, is superior, which leads to a uniform sequencing depth and diagnostic coverage.

As described, GS opens up the possibility of recognizing complex variant types that can only be captured to a limited extent with ES (SVs, REs). It has been suggested that in order to maximize the sensitivity of detection, pipelines should use several variant detection tools (Table 2), i. e., tools that are specific to each variant class (24). Combining the results of two orthogonal SNV/InDel callers (e. g., GATK HaplotypeCaller, DeepVariant, Strelka2), and then merging multiple variant callsets (in VCF format) into a single callset using software packages such as BCFtools can provide a slight sensitivity advantage. However, care should be taken to ensure correct handing of complex variants and/or differences in variant representation (8).

**Table 2:** Frequently used software modules for variant calling, quality checking, and other processing steps necessary in short-read sequencing. (Information on the named software is available upon request.)

**Analysis step**	**Processing step**	**Software (example)**
Primary analysis	Demultiplexing	bcl2fastq
	Alignment	BWA-Mem, DRAGMAP
	File management	BCFtools
	SNV calling	GATK HaplotypeCaller, DeepVariant, Strelka2
Secondary analysis	CNV calling	MOPS, CNVkit, CONTRA, CoNVEX, ExomeCNV, ExomeDepth, GATK, XHMM
	Structural Variant calling	DELLY, Lumpy, Manta, Pindel, SVMerge
	Repeat expansions	ExpansionHunter, exSTRa, STRetch, TREDPARSE
	TEs and insertions	Mobster, MELT, TraFiC-mem
	Low frequency variants (mosaic and somatic)	MuTect2

The laboratory should validate both the impact of the aligner and the accuracy of variant detection for all detectable variant classes, i. e., for SNVs, InDels, CNVs, and complex genomic structural variants (SVs, REs) separately (EuroGen recom. 22 (6)). Sequencing of the whole genome represents the most comprehensive approach, which typically yields an average sequence depth of around 30–60x across the entire genome. However, the higher sequence depth achieved in panel- and exome sequencing may allow a more sensitive detection of low allelic frequency variants, e. g., subclonal somatic mutations in tumors or variants that present as germline mosaics (24). The reliability and accuracy of algorithms for the detection of SVs and REs from GS data remain suboptimal at present. However, they allow the detection of a large number of pathogenic changes, and currently tend to be used as a screening method. Before reporting such complex variants, technical confirmation may be required using an alternative, diagnostically established method.

##### 3.5.2.3 Tertiary analysis

The first step in tertiary analysis is variant annotation, which involves the naming of each variant using a standardized nomenclature, followed by the linking it to information from various databases, the medical literature, and information concerning the quality of variant detection (e. g., the number of reads in which the variant occurs). Here, the nomenclature guidelines for SNVs from the Human Genome Variation Society (HGVS), and the nomenclature guidelines for CNVs from the International Standing Committee on Human Cytogenetic Nomenclature (ISCN), should be used (Table 3). Correct annotation is required for the subsequent filtering, prioritization, classification, and interpretation of variants (see 3.6). For complex genomic variants, such as multinucleotide variants (MNVs)—which are usually listed independently, but which must be considered jointly in order to allow correct assessment of their pathogenicity—annotation remains challenging. The correct detection and annotation of complex structural variations, such as inversions and balanced translocations, can also be difficult using GS data alone, and may require the use of an additional method.

**Table 3:** Further information on selected nomenclature guidelines and reference materials.

**Name**	**Website**
Human Genome Variation Society (HGVS)	https://varnomen.hgvs.org/
Human Gene Nomenclature (HUGO)	https://www.genenames.org/
Matched Annotation from the NCBI and EMBL-EBI (MANE)	https://www.ncbi.nlm.nih.gov/refseq/MANE/
International Society of Cytogenetic Nomenclature (ISCN)	https://iscn.karger.com/
Genome-in-a-bottle	https://www.nist.gov/programs-projects/genome-bottle

EuroGen recommends that all GS variants identified at a given site should be annotated (EuroGen recom. 23), and that the aggregated data should be compiled in order to generate internal allele frequencies and allow the interpreted data to be shared with other institutions (EuroGen recom. 24 (6)).

##### 3.5.2.4 Pipeline quality control and validation cycles

The bioinformatics pipeline should have sufficient quality control measures to ensure that the generated data are robust, and that error detection and compliance are provided (EuroGen recom. 16, 21 (6)). To enable quality control of the bioinformatics process, quality data can be used in each step of the analysis (Table 4). The NGS pipeline should be platform-specific and tailored to the needs of the laboratory (EuroGen recom. 17 (6)).

**Table 4:** Relevant quality parameters in the NGS process that are also recorded within the framework of the EMQN Quality assessment schemes.

**Parameter**	**Definition**
Yield	Throughput of the sequencing run.
Error Rate	Percentage of mismatched bases per cycle, as determined by alignment to PhiX.
%Q30	Percentage of bases identified with a quality score of 30 (equivalent to 1 error in 1000 bases, Phred score) or above.
Density (K/mm2)	Cluster density in thousands per mm2.
Cluster PF (%)	Cluster densities that satisfy the internal chastity filter and can therefore be used for downstream analysis.
Phas/Prephas (%)	Percentage of molecules in a cluster that gets out of sync in each cycle due to incorporation of multiple nucleotides (pre-phasing) or due to failed incorporation of a nucleotide (phasing).
Percentage mapped reads	Percentage of reads that could be mapped to the reference sequence.
Duplicates (%)	Percentage of duplicates not typically used for downstream analysis.
Insert Size	Average size of sequenced DNA molecules.
GC content (%)	Average GC content of the sequenced DNA fragments.
Mean Coverage	Average number of reads covering a specific target region.
Cov30	The average number of bases sequenced that match or “cover” known reference bases. For example, an entire genome sequenced with 30-fold coverage means that each base in the genome has been sequenced an average of 30 times.
Uniformity	Coverage uniformity describes the distribution of reads across the target regions in the genome.

Bioinformatic pipelines developed for the analysis of GS/ES clinical data are complex and require a robust quality assurance program for ongoing monitoring and pipeline updates. Validations must document all steps required to compile, install, and run specific GS clinical bioinformatic pipelines. Both a version control system and the detailed documentation of code changes are required for the deployment of all software updates ((25); IEC62304).

Pipelines should be tested against reference standards (e. g., genome-in-a-bottle samples) to ensure they are operating in a reproducible and error-free manner (26). Validation is performed on a specific version of the software, and the data analysis should be repeated whenever software components or reference data files are updated. Laboratories should have a Release Management process, with a clear definition of “major” and “minor” changes that define the level of validation. If relevant changes are made to software components or parameters, the testing mechanism should revalidate both the local test performance and the impact of these changes on the clinical process of variant detection and annotation. Ideally, software validation should include continuous integration processes for updates and enhancements. For incremental changes, software testing may need to be automated to some extent. Crucially, errors must be tracked down effectively, and underlying issues must be identified and resolved.

#### 3.5.3 NGS quality parameters

Important quality parameters (Table 4) for NGS analysis should be recorded for each run using tools such as fastQC, multiQC, or the Illumina Sequence Analysis Viewer (SAV). During the analysis, care should be taken to consider—as far as possible—the metrics per read and per lane, since a decrease in quality in a certain area of the flow cell, for example, can be overlooked in a global run statistic.

Depending on the method and application, acceptance limits for individual values may differ. For example, for ES applications, an average target region coverage of 100–120X should be aimed for, while 30–40X may be sufficient for GS. From the perspective of diagnostic quality, coverage of the target regions with a defined minimum coverage (e. g., 20X) that allows reliable detection or exclusion of a change is also crucial. In terms of the target region, a distinction should be made between the target region that is used for assessing the assay (e. g., target region for enrichment) and the target region of diagnostic relevance (e. g., consensus coding sequence (CCDS) plus/minus 10 bp). In PCR-free GS analyses, values of >99 % with a coverage of at least 20X are typically achieved for the autosomes. The degree to which a deviation from the set quality cutoffs influences the quality of the analysis result must be evaluated within the context of the clinical question and in accordance with the intended application.

In the case of specific questions, e. g., with regard to mosaic evaluation and tumors, further quality assurance considerations, such as those relating to sequencing depth, reported allele frequencies, and tumor-specific variant evaluation, may be necessary.

### 3.6 Post-analytics

This issue is defined by ISO 15189, and stipulates, among others, the requirements for the release of test results. In addition to the aforementioned criteria for the evaluation of results, post-analytics should include the plausibility check, e. g., in order to allow the collection of additional information using alternative methods (e. g., Sanger sequencing, Multiplex Ligation-dependent Probe Amplification/MLPA) when the examination results are not sufficiently reliable in terms of the test request.

#### 3.6.1 Variant evaluation

Variant assessment is one of the core competencies of human geneticists, as it represents the basis for further counseling and (personalized) therapy for patients with genetic diseases. Due to the central importance of variant evaluation in terms of patient care, evaluation of the detected genetic changes must be comprehensibly guaranteed via a uniform standard procedure. Variant assessment should be based on the five-class system developed by the American College of Medical Genetics and Genomics (ACMG)/Association for Molecular Pathology (AMP) in 2015 (27): benign, probably benign, variant of unclear significance (VUS), probably pathogenic, and pathogenic. Many laboratories use the classification system of the ACMG, and categorize variants into classes 1 (benign) to 5 (pathogenic). However, this classification is not used internationally, and may lead to misunderstandings. The report must provide a reference for the classification used.

In the ACMG classification, a total of 16 pathogenic and 12 benign criteria are used for classification, all of which differ in terms of their respective level of evidence. In recent years and based on these five classes, multiple recommendations for the application and interpretation of individual ACMG/AMP criteria have been developed. Here, the recommendations for variant classification of the “Association for Clinical Genomic Science” (ACGS)(https://www.acgs.uk.com/media/11631/uk-practice-guidelines-for-variant-classification-v4-01-2020.pdf) should be emphasized. Among others, these make suggestions as to how the individual criteria can be used and combined and can indicate a possible upgrading or downgrading of the level of evidence for the respective criteria. These recommendations should be included to ensure correct variant evaluation. Further important general recommendations on evidence criteria that should be taken into consideration are those developed by the Clinical Genome Resource (ClinGen) Sequence Variant Interpretation Working Group (SVI WG) (https://clinicalgenome.org /working-groups/sequence-variant-interpretation/). For some criteria, a point-based system is proposed, which should be used to facilitate decision-making aid when evaluating individual levels of evidence.

The use of suitable databases is essential for correct variant evaluation. Besides general variant-, population-, and sequence databases, most of which are already listed in the 2015 ACMG/AMP publication, a number of other gene or phenotype-specific databases have been established that should be consulted for specific indications (Table 5). In addition, the gene-specific guidelines of the “Variant Curation Expert Panels” (VCEP) from ClinGen, in which the interpretation and application of individual evidence criteria were explicitly determined by a specialist committee, should be taken into account when evaluating variants in the respective genes. An overview of this can be found in the “ClinGen Criteria Specification” (CSpec) Registry (https://cspec.genome.network/cspec/ui/svi/). The VCEP assessments, and the assessments of some variants from gene or phenotype-specific databases, are now available in general databases such as ClinVar and LOVD (Table 5).

**Table 5:** Selection of databases that are generally used for variant assessment but which also contain data on specific genes.

**Variant databases**	**Content or specifically recorded genes and phenotypes**	** Website**
**ClinVar**	Genome wide	https://www.ncbi.nlm.nih.gov/clinvar/
**LOVD**	Genome wide	https://www.lovd.nl/
**HGQN**	Genome wide	https://www.hgqn.org/
**DECIPHER**	Genome wide	https://www.deciphergenomics.org/
**BRCA Exchange (ENIGMA)**	*BRCA1, BRCA2*	https://brcaexchange.org/?=
**CanVar**	Tumor predisposition syndromes	https://canvaruk.org/
**CFTR1/CFTR2**	*CFTR*	http://www.genet.sickkids.on.ca/Home.html https://cftr2.org/resources
**DVD**	Hearing impairment	https://deafnessvariationdatabase.org/
**Emhg**	*RYR1, CACNA1S*	https://www.emhg.org/diagnostic-mutations
**Infevers**	Autoinflammatory Diseases	https://infevers.umai-montpellier.fr/web/index.php
**InSiGHT**	*APC, CDH1, EPCA, GALNT12, MLH1, MSH2, MSH6, MUTYH, PMS2*	http://insight-database.org/
**MITOMAP**	Mitochondrial Diseases	https://www.mitomap.org/MITOMAP
**PKD**	*PKD1, PKD2*	https://pkdb.mayo.edu/variants
**RettBASE**	*MECP2, CDKL5, FOXG1*	http://mecp2.chw.edu.au/
**Population databases**		
**gnomAD**	Genome wide	https://gnomad.broadinstitute.org/
**dbSNP**	Genome wide	https://www.ncbi.nlm.nih.gov/SNP/
**DGV**	Non-pathogenic structural variants	http://dgv.tcag.ca/dgv/app/home
**MitoPhen**	Patients with pathogenic mtDNA alteration	https://www.mitophen.org/
**Sequence databases**		
**NCBI nucleotide database**	Compilation of sequences from different databases	https://www.ncbi.nlm.nih.gov/nucleotide/
***TTN*-Transcript**	Locus reference genome for *TTN*	https://www.cardiodb.org/titin/index.php

Besides being used for the evaluation of variants in the laboratory, databases such as the ClinVar and LOVD, as well as the newly established HGQN (Human Genetics Quality Network, BVDH database), are also used to make newly detected variants publicly accessible. In this way, knowledge concerning the possible pathogenicity of both new and established variants can be shared.

Due to the complexity of variant assessment, numerous recommendations have been made in recent years with regard to evidence criteria, and in the future, more specifications are to be expected. A point system for determining the five classes has already been discussed (28). In the future, the authors advice that evaluation of genetic changes should be carried out by specialists in human genetics in order to ensure that the relevant criteria and recommendations are applied correctly and to guarantee harmonization of variant evaluation against the background of the yet to be expected complexity. A prerequisite for this is documentation of the standardized procedure for variant evaluation within the respective facility. Harmonization can also be facilitated via continuous education, e. g., consensus training.

#### 3.6.2 Data Storage

Various issues must be taken into account with regard to the requirements for data storage in NGS processes. These relate to both legal and normative requirements, as well the in-house policy of the respective facility.

As already explained, genome diagnostics is subject to the same legal requirements as all other forms of human genetic diagnostics. In particular, NGS data storage must adhere to the general medical obligation to retain patient documents for ten years after cessation of treatment (Bürgerliches Gesetzbuch (BGB), § 630 f BGB) and the regulations of GenDG (§ 12). The latter stipulates that the results of genetic examinations and analyses must be stored in the case notes of the respective individual for ten years. The obligation to destroy the results of genetic investigations and analyses, which is also formulated in this legislation, is discussed in detail elsewhere by S. Heidemann (29).

In general, the documentation requirements specified in ISO 15189 must be taken into account, particularly with regard to datasets in the sense of medical records (ISO 15189: 4.13). For example, these must be easily accessible and protected against unauthorized changes, and in the event of authorized changes, both the time at which the change is made and the individual who makes the change must be documented.

Therefore, a facility that offers NGS analyses must formulate an in-house policy for NGS data storage. In addition to the above-mentioned legal requirements, this must take into account matters such as technical and content-related issues, requirements for the fulfilment of accreditation standards, and the further eventual use of the data to address scientific questions.

In particular, decisions concerning the extent of data storage should take into account the degree to which the available and standardized software modules for data processing and compression can lead to an incorrect evaluation, and whether a loss of information of relevance to evaluation and interpretation can occur. If this evaluation is negative, the storage of VCF files may be sufficient. However, the issue of whether the storage of other file formats is, or could become, necessary, e. g., within the research context, must be considered. Storage of NGS data as a CRAM file represents one of the most space-efficient forms. These files contain both the positions of the individual sequences in the genome, and almost all of the information of the FASTQ files. Only information regarding raw data filtering, control number (which is no longer used with modern sequencers), and the index sequence, all of which is stored in the read name, is lost. Furthermore, in contrast to the FASTQ format, information related to Read 1 or 2 is saved. If UMIs (unique molecular identifiers) are used, they must be stored in the metadata, since all information encoded in the read name is lost during compression. Compared to FASTQ and BAM files, the conversion to CRAM format can reduce file size by up to 60 %, depending on the desired degree of compression, with or without data loss. In addition, in contrast to FASTQ files, BAM and CRAM files allow the storage of additional metadata, and it is thus possible, for example, to include the patient ID directly in the file. Increasingly, CRAM files are becoming the preferred data storage format. However, the software solutions selected for secondary and tertiary analysis must be capable of handling this format efficiently. Therefore, many laboratories first convert CRAM files to BAM file format, and reserve the use of CRAM files for archiving purposes only (30).

Against this background, the ISO 15189 stipulates that the laboratory must formulate regulations for the storage of NGS data sets. The saving of variant tables/VCF files may be sufficient, since the evaluation and interpretation steps that contribute to the generation of results (as defined by the GenDG) can be traced back to the variant tables. However, it should be noted that the restriction of storage to VCF files only renders the checking of variants at a later time-point impossible, e. g., with regard to the presence of artifacts, such as alignment errors. A visual inspection of variants, e. g., using the IGV Viewer, is therefore no longer possible. This can represent a limitation in terms of the later re-analysis of NGS data.

Should a new analysis of raw data be required, in principle, a new NGS analysis (wet laboratory, data processing)—if necessary with a repeat sample collection—is suitable in order to incorporate further methodological developments in NGS diagnostics. However, this is not always possible. Thus, every institution should consider archiving the sequence data in the form of FASTQ, BAM, or CRAM files.

### 3.7 Reporting of findings

The structure and content of reports are based on the requirements of ISO 15189 and the S2k guideline for human genetic diagnostics (20). In addition to information on patient identification and the indication for the test, the first page should include a concise description of the result that is of relevance to the sender in terms of diagnosis, prediction, therapy, or genetic counseling (EuroGen recom. 38 (6)). As with other reports of findings from genetic analyses, the method must also be clearly described. This description should include all of the essential evaluation parameters, in particular information on the test procedure; the variant types that can be detected with the method; the coverage and reading depth of the target region (region of interest), i. e., the exome, genome, and/or the genes that are reported explicitly; as well as analytical sensitivity and precision (EuroGen recom. 37 (6)). The report should also include details of the reference genome, reference transcript version, and OMIM reference (EuroGen recom. 39, 40 (6)), as well as information on variant assessment (e. g., ACMG). The versions proposed by the MANE initiative should be used as reference transcripts. The extent to which database and literature references, as further explanations of the clinical relevance of (probably) pathogenic variants, are mentioned in the findings is to be defined by the laboratory. While all variants that are pathogenic or probably pathogenic according to the ACMG classification and that are of relevance to the indication for testing are reported, VUS should only be evaluated and reported if they are located in genes that are clearly associated with a known phenotype (EuroGen recom. 42 (2, 5, 6)).

### 3.8 Quality assurance measures

The laboratory must select, and then validate or verify, examination methods that are suitable for clarifying the given medical question. Guidelines and notes for validating NGS-based examination methods are available (2, 5, 6). For GS, special supplementary recommendations have been published (21). The validations of the platform and the pipeline should consider reference materials such as genome-in-a-bottle samples (GiaB, Tab. 4). The use of gene panels to clarify specific questions implies validation at the gene level. The coverage of known disease-causing variants should be ensured, and possible limitations of the methodology (e. g., pseudogenes) should be taken into account. Transcripts that have been harmonized across databases should be used as reference sequences (3).

In terms of sample tracking, genotyping should be established using an orthogonal method that is used to match SNVs (EuroGen recom. 29 (6)), if sample tracking is not secured in any other manner. As a minimum, quality control of the analyses should include monitoring of the coverage determined by the laboratory.

In the case of prenatal analyses, the exclusion of maternal contamination is a further mandatory quality assurance measure.

Quality assurance also includes participation in external quality assessment (EQA) schemes (proficiency testing, interlaboratory comparisons) which are able to assess whether or not the intended level of quality has been achieved. The requirements for this are specified in the new version of RiLiBÄK, which is currently being drafted. ISO15189 sets common specifications for quality assurance. The laboratory must select EQA schemes that cover all clinically relevant issues and which review the entire analysis procedure. This includes consideration of wet laboratory procedures, data processing, variant classification and interpretation, and reporting. Corresponding EQA schemes are offered by bodies such as BVDH, EMQN, UK NEQAS, and GenQA.

## 4 Summary

In general, quality assurance measures in genome diagnostics follow the general guidelines formulated for human genetic diagnostics. However, the complexity of data processing, storage, and variant evaluation within genome diagnostics, as well as the ongoing technical developments, require specific in-house regulations that go beyond the usual specifications formulated, for example, by RiLiBÄK and ISO 15189. Some of these issues should be made known to the sender in advance of the analysis, but if necessary, they must also be discussed when reporting the results. The implementation of quality assurance measures is the responsibility of the genetic testing facilities. Formal competence according to ISO 15189 can be assessed and monitored by external organizations (e. g. German Accreditation Body DAkkS). A continuous adaptation to ongoing developments guarantees state-of-the-art diagnostics, which serves in turn as the basis for personalized medicine in the multidisciplinary context.
